# Selective use of percutaneous testis biopsy to optimize IVF-ICSI outcomes: a case series

**DOI:** 10.1186/s40738-016-0020-y

**Published:** 2016-04-15

**Authors:** Russell P. Hayden, Diane L. Wright, Thomas L. Toth, Cigdem Tanrikut

**Affiliations:** 1 Department of Urology, Massachusetts General Hospital, Boston, MA USA; 2 Inception Infertility, Houston, TX USA; 3 Department of Obstetrics & Gynecology, Massachusetts General Hospital, Boston, MA USA; 4 MGH Fertility Center, 55 Fruit Street, YAW 10A, Boston, MA 02114 USA

**Keywords:** IVF, ICSI, Male factor infertility, TESE, Kartagener syndrome

## Abstract

**Background:**

Sperm quality may degrade during transit through the male reproductive tract in some individuals. In this setting surgically retrieved testicular sperm may outperform ejaculated samples for use with in vitro fertilization (IVF) and intracytoplasmic sperm injection (IVF-ICSI). We sought to describe one center’s experience with the use of fresh testicular sperm after prior failed IVF-ICSI with ejaculated samples.

**Results:**

A retrospective review was conducted evaluating IVF-ICSI cycles performed at a tertiary IVF unit between 2009 and 2014. Couples who were managed with percutaneous testis biopsy to obtain sperm, despite availability of ejaculated sperm, were included. Four couples who underwent a total of 6 percutaneous testis biopsy/IVF-ICSI cycles were identified. Collectively, the couples had undergone 9 prior IVF-ICSI cycles using fresh ejaculated sperm without successful pregnancy. From the six cycles that used fresh testicular sperm four live births resulted (1 twin gestation, 3 singletons). Only 1 of the 4 couples remained childless.

**Conclusions:**

For patients who have had prior failed IVF-ICSI attempts, this small case series demonstrates a possible therapeutic benefit when freshly procured testicular sperm are used in lieu of ejaculated samples.

## Background

With the advent of in vitro fertilization (IVF) and intracytoplasmic sperm injection (IVF-ICSI), many men with severe male factor infertility can now be offered the opportunity to parent their own genetic offspring. Unfortunately, a subset of patients will not succeed despite these advanced treatments. Although the chance of a live birth continues to rise with an increasing number of IVF cycles, up to 19 % of couples will remain childless following 8 attempts [1]. Given the financial, physical, and psychological demands each IVF cycle requires [2], a significant drop-out rate accrues with each successive treatment [3].

For some couples with multiple failed IVF cycles, sperm-related factors may play a principle role. Unfortunately, robust indicators of sperm quality have yet to be identified. Most centers routinely select for sperm based upon visual cues such as motility and morphology. Fresh ejaculated sperm, when available, are generally preferred over surgically retrieved samples given the need for an invasive procedure. These practice patterns are often based upon the belief that immature testicular sperm may be less likely to result in a successful pregnancy. However, this tenet is increasingly being challenged as IVF-ICSI outcomes correlate poorly with traditional sperm quality parameters [4, 5]. Studies involving large cohorts have now shown near equivalence with samples retrieved from the epididymis or testis [6–8], and even with sperm characterized by poor motility and morphology [5]. In the extreme scenario of substantial necrospermia, testicular sperm may even outperform viable ejaculated samples [9].

Investigators have begun to focus on more sophisticated rubrics of sperm quality in order to understand and optimize IVF-ICSI outcomes. Reactive oxygen species (ROS) and DNA damage have been two areas of intense investigation. Preliminary research has also shown that sperm quality, especially in terms of DNA integrity, may decrease in some patients as the spermatozoa transit through the male reproductive tract [10–12]. As a result, some couples may benefit from testis-derived sperm even in the presence of viable sperm in the ejaculate.

Although it is difficult to rule out an occult sperm-related etiology when evaluating a couple with recurrent IVF failure, an attempt with testicular sperm is an option rarely offered when ejaculated sperm are available. The following case series explores the premise that a subset of couples may benefit from freshly procured testicular sperm in an attempt to bypass male factors related to post-testicular events.

## Methods

IRB approval was obtained for this investigation. A retrospective chart review was conducted to evaluate all IVF-ICSI cycles at a tertiary referral center. Records from January 2010 to January 2014 were examined. Inclusion criteria consisted of couples who were managed with percutaneous testis biopsy (PercBx) for any indication at our institution. A total of 19 consecutive cases were identified. Men who had obstructive azoospermia as the indication for PercBx were excluded from the study population, which resulted in four unique couples (Table 1) who underwent a total of six IVF-ICSI treatment cycles using freshly retrieved testicular sperm (Table 2). Cryopreservation was not included in our treatment protocol to avoid diminished sperm yields from the freeze-thaw cycle, and to avoid the theoretical risk of reduced implantation rates [13].

**Table 1:** Couples’ clinical characteristics

	Number of prior cycles	Male		Female	
		Age	Infertility diagnoses	Age	Infertility diagnoses
Case 1	4	34	OAT^a^	31	None
			Elevated DFI^b^		
Case 2	1	49	OAT	37	Advanced Maternal Age
					Corrected Uterine Factor
Case 3	3	32	Kartagener’s Syndrome	32	Corrected Uterine Factor
Case 4	1	46	OAT	38	Advanced Maternal Age
			Elevated DFI		Decreased Ovarian Reserve

^a^Oligoasthenoteratospermia, ^b^DNA fragmentation index

**Table 2:** IVF-ICSI cycle outcomes using testis-derived sperm obtained via percutaneous biopsy

	Number of oocytes (number mature)	Number fertilized (%)	Number of 6–10 cell embryos by day-3	Day-3 embryos transferred	Pregnancy outcome
Case 1	13 (11)	5 (45)	3	8 cell, 7 cell	Singleton Delivered
Case 2	16 (11)	5 (45)	2	8 cell, 6 cell	Singleton Delivered
Case 3	14 (13)	9 (69)	7	10 cell, 8 cell	Singleton Delivered
	17 (16)	7 (43)	2	10 cell, 8 cell	Twins Delivered
Case 4	5 (3)	0 (0)	NA	NA	NA
	3 (3)	2 (67)	2	7 cell, 6 cell	Terminated Ectopic

All couples had undergone at least one unsuccessful IVF-ICSI cycle using a fresh ejaculated sample (mean 2.25 cycles) prior to proceeding to IVF-ICSI with PercBx. Female partners were evaluated by a reproductive endocrinologist and optimized for another IVF-ICSI cycle. Each male partner underwent thorough urologic evaluation and informed consent prior to proceeding with percutaneous testis biopsy. Semen analyses were interpreted according to the 2010 WHO guidelines. Normal testis volume was considered > 15 mL. All patients underwent a basic endocrine work-up, including follicle stimulating hormone (FSH, lab normal < 12 mIU/mL), and morning total testosterone (normal > 270 ng/dL).

All sperm procurements were coordinated on the day of oocyte retrieval. Male evaluations and testicular biopsies were performed by a single reproductive urologist (CT). After initiating local anesthesia via a spermatic cord block an 18-gauge hollow core biopsy needle was used to percutaneously procure 4 to 10 cores of testicular parenchyma. An embryologist provided immediate feedback to confirm the presence of adequate numbers of sperm for the anticipated number of oocytes. Unilateral biopsy was sufficient in each circumstance.

## Results

### Case 1

The couple presented with a history of 4 failed IVF-ICSI cycles in the setting of known oligoasthenoteratospermia (OAT). Further testing revealed an elevated sperm DNA fragmentation index (DFI). The female partner was 31 years old with an unremarkable gynecologic evaluation.

Her partner was a healthy 34-year-old nonsmoker with no prior conceptions or conception attempts. On physical exam the patient was found to have symmetric 27 cc testes. There was no palpable varicocele. His FSH was 1.2 mIU/mL and his morning testosterone was 420 ng/dL. Four semen analyses were available and remarkable for variable OAT, with concentrations 0.1 – 27 million/cc, progressive motility 0 – 25 %, and Kruger morphology 0 – 1 % normal forms. Total motile counts ranged from 0.3 to 13 million. His karyotype was normal.

The couple’s four prior IVF-ICSI attempts utilized ejaculated sperm. Each cycle resulted in the retrieval of 8 to 14 mature oocytes. The first cycle resulted in an early spontaneous abortion. The second attempt yielded no fertilized oocytes. Fertilization rates of the third and fourth cycles were 44 % and 64 % with 1 and 2 embryos transferred, respectively. Neither cycle resulted in pregnancy. Given the couple’s history of 4 failed IVF-ICSI cycles a sperm chromatin structure assay (SCSA) was ordered, noting an elevated DFI of 44.2 %.

In light of four unsuccessful IVF-ICSI cycles and indicators of poor sperm quality, including elevated DFI, the couple was counseled to consider an attempt using a fresh testicular sperm extraction at the time of oocyte retrieval. The couple chose to pursue this option; eleven mature oocytes were retrieved, all of which were injected with fresh testicular sperm. Five oocytes fertilized with two day three embryos (8 cell, 7 cell) transferred. A resulting healthy singleton pregnancy was delivered.

### Case 2

The couple presented with one failed IVF-ICSI cycle marked by poor embryo development in the context of cryptozoospermia following vasectomy reversal. The female partner was 37 years old with no prior history of conception. Her evaluation was notable only for a small endometrial polyp that was resected. Follow-up transvaginal ultrasound confirmed a normal uterine cavity.

Her partner was a 49-year-old nonsmoker with one prior spontaneous conception with another partner prior to his vasectomy 12 years earlier. He had undergone a vasectomy reversal 4 years after initial vasectomy. On exam his testes were 27 cc in volume, and there were no palpable varicoceles. Although he initially had return of motile sperm to the ejaculate (1 – 8 million total motile sperm per sample), his semen analyses demonstrated a progressive deterioration of motile counts prior to referral to our center. Five preoperative semen analyses were available. At the time of treatment samples required centrifugation with fewer than 20 immotile sperm obtained per pelleted sample. His hormonal profile was otherwise reassuring with an FSH of 2.0 mIU/mL and a morning serum testosterone of 423 ng/dL.

The couple’s initial IVF-ICSI attempt utilized rare immotile sperm from an ejaculated sample identified only after several hours of searching by two embryologists. A total of five mature oocytes were retrieved and two fertilized. Both embryos arrested at the 2-cell stage prior to a transfer attempt.

Due to the degree of cryptozoospermia and poor embryo development evident with the first cycle attempt, the couple opted to pursue a second cycle using freshly-retrieved testicular sperm. Twitching sperm were obtained and five of 11 mature oocytes fertilized normally. Two high quality embryos (8-cell, 6-cell) were transferred on day three. A singleton pregnancy resulted with successful term delivery of a healthy male infant.

### Case 3

The third couple was characterized by multiple failed IVF-ICSI cycles using fresh ejaculated immotile sperm in the setting of known Kartagener’s Syndrome in the male. The female partner was 32 years old with no prior conceptions or attempts. She had a history of symptomatic mural fibroids for which she underwent laparoscopic myomectomy without complication. Subsequent office hysteroscopy confirmed a normal uterine cavity. Her testing and evaluation were otherwise unremarkable.

Her partner was a 32-year-old nonsmoker who was diagnosed with Kartagener’s Syndrome at the age of three. On exam his testes were symmetric and 32 cc’s in volume, and there were no clinical varicoceles. His endocrine workup revealed an FSH of 4.9 mIU/mL and a morning serum testosterone of 437 ng/dL. Semen analyses, as expected, demonstrated a complete absence of motility. Over the course of his evaluation, six semen analyses were performed. Concentrations ranged from 8 to 19 million/cc (total count 16 to 38 million) with 0 to 2 % normal forms by strict criteria.

Following the female partner’s myomectomy and post-operative recovery, the couple had undergone three IVF-ICSI attempts using fresh ejaculated sperm. Each cycle resulted in the retrieval of 4 to 12 mature oocytes, with resulting fertilization rates of 0 to 42 %. A single blastocyst was transferred during the first cycle without success. The second and third cycles utilized sperm selected based upon the hypo-osmotic swelling assessment in an effort to optimize outcomes. Viability testing was also judged with the mechanical touch technique, which uses a combination of morphology and tail flexibility for assessment [14]. Two day three embryos were transferred with the third cycle resulting in a biochemical pregnancy that did not progress.

Following the three failed cycles, the couple elected for another attempt using fresh testicular sperm. This cycle resulted in the retrieval of 13 mature oocytes, nine of which fertilized. Two high quality embryos (10-cell, 8-cell) were transferred on day three. The couple successfully delivered a singleton pregnancy at term. A single resulting blastocyst was also frozen.

The couple desired further family building and pursued transfer of their remaining frozen blastocyst. Unfortunately no pregnancy resulted, and the couple proceeded to another IVF-ICSI attempt using freshly procured testicular sperm. With this cycle, seven of 16 mature oocytes fertilized normally and two day three embryos were transferred (8-cell, 10-cell). A resulting twin pregnancy was successfully delivered.

### Case 4

The final couple presented with both decreased ovarian reserve and OAT in combination with an elevated sperm DFI. The woman was 38 years old when she initiated treatment at our center. A previous fertility evaluation had demonstrated a day three FSH of 10.8 (normal < 9) and an antral follicle count of 9 (ideally > 14). The remainder of her workup was unremarkable.

Her partner was a 46-year-old nonsmoker who had a history of vasectomy 15 years prior, which was then reversed after a 14 year obstructive interval. He initially had return of motile sperm to the ejaculate with progression over time to OAT. His sperm concentrations dropped from a high of 9 to 0.3 million/cc, presumably due to partial anastomotic stenosis. At the time of our evaluation, by now his fifth semen analysis, sperm concentration had further declined to 0.1 million/cc with 25 % progressive motility and 3 % normal morphology (<1 million total motile sperm). The patient otherwise had a reassuring hormonal profile, with an FSH of 3.8 mIU/mL and a morning serum testosterone of 474 ng/dL. His exam demonstrated 30 cc testes bilaterally with a small left varicocele.

The couple’s initial IVF-ICSI attempt using fresh ejaculated sperm resulted in the retrieval of 6 mature oocytes, only one of which fertilized. The resulting 5-cell embryo was transferred on day three and ended in biochemical pregnancy without progression. An SCSA was subsequently obtained demonstrating an elevated DFI of 42.8 %.

Given the elevated DFI, poor fertilization rate, and unsuccessful outcome of their first cycle, the couple chose to proceed with another IVF-ICSI cycle using fresh testicular sperm. Two subsequent testicular sperm extraction cycles were conducted: the first IVF cycle resulted in the retrieval of three mature oocytes, none of which fertilized following injection with motile sperm. With the second cycle a total of three mature oocytes were retrieved, two of which fertilized. Both embryos were transferred on day three (6-cell, 7-cell). An ectopic pregnancy resulted and was managed medically. Afterward, the couple elected to stop further IVF attempts.

## Discussion

The sperm-related factors that result in limited fertilization, poor embryo development, impaired implantation, and early spontaneous abortion are poorly understood. Historically, sperm motility and morphology have been the primary surrogates for sperm quality; early studies substantiated this concept in terms of poorer male factor-related outcomes using IVF with conventional insemination [15–17]. However, the predictive values of morphology and motility have been questioned when the standard insemination process is bypassed [18, 19]. In an effort to better characterize sperm quality in the era of IVF-ICSI, investigations into more sophisticated measures of sperm quality such as oxidative damage and impaired DNA integrity have been pursued.

A relationship between oxidative stress and subfertility has now been established. Elevated seminal ROS are negatively correlated with sperm concentration, motility, and morphology [20, 21]. Additionally, elevated ROS are found in men who have undergone vasectomy reversal [22]. Cases 2 and 4 are characterized by OAT in men who were previously treated with vasovasostomy. It is unclear if elevated ROS played a dominant role in these cases, although their presentations can be considered consistent with oxidative stress.

A direct link between sperm oxidative damage and embryo demise has been demonstrated in animal models [23]. Additionally, elevated seminal ROS are correlated with poor embryo development in humans [24]. Relatively little is known regarding the accumulation of oxidative damage as sperm progress through the male reproductive tract. Ollero et al. postulated that the presence of immature spermatozoa, a known source for endogenous ROS [25], may result in damage to mature sperm during transit through the epididymis [26]. The local environment within the epididymis may be further stressed as a vasovasostomy site begins to stenose. As pressure builds within the testicular side of the anastomosis, progressive epididymal dysfunction may ensue, particularly in a system that has suffered iatrogenic obstruction in the past [27]. The use of testicular sperm may bypass this potentially toxic local environment, and may translate into improved IVF-ICSI outcomes in patients with similar profiles as Cases 2 and 4.

A known result of oxidative stress is DNA damage, which is a prominent feature of Cases 1 and 4. For both patients an SCSA revealed a DFI above 40 %. In one of the largest series by Bungum et al., a DFI greater than 30 % was found to translate into an odds ratio of 0.1 (CI 0.02 – 0.42) for clinical pregnancy rates following intrauterine insemination [28]. Their data found no statistical difference for their conventional IVF and IVF-ICSI cohorts, which was initially attributed to selection bias regarding oocyte quality. A subsequent meta-analysis corroborated these findings regarding IVF-ICSI and an elevated DFI, although a trend toward a higher miscarriage rate was noted [29]. Despite the fact that IVF-ICSI outcomes remain favorable even in states of marked DNA damage [30], conceivably due to the DNA repair capacity of high quality oocytes [31], the possibility remains that a subset of patients may be particularly affected by an elevated DFI. Greco and colleagues examined a small cohort of couples who were similar to Case 1: patients who were characterized by an elevated DFI and recurrent IVF-ICSI failure using ejaculated sperm [11]. They found that testis-derived samples had significantly less DNA damage. Although fertilization rates and embryo development were no different in the testis versus ejaculated samples, the testicular sperm achieved eight clinical pregnancies as opposed to one in the control group. They postulated that significant DNA damage accrues during transit though the epididymis, similar to the concept raised by Ollero et al. [26].

Some investigators have attempted to treat impaired DNA integrity with antioxidants in order to limit oxidative stress. Abad et al. demonstrated a modest improvement in semen parameters and DFI following 3 months of oral antioxidant therapy in a group of infertile men [32]. In the case of testicular derived sperm, a small case series by Greco et al. found improved ICSI outcomes when men with poor DNA integrity are pretreated with oral antioxidants [33]. A summative review by Kumalic and Pinter substantiated the beneficial effects of antioxidant therapy, albiet most of these results are based upon ejaculated samples [34]. In our practice we routinely offer men with asthenospermia supplementation with coenzyme Q10. It remains unclear if the outcomes would have differed if the men in this case series were offered a formal course of antioxidant therapy. Further studies are needed to expand upon the preliminary results of Greco et al., in which testicular sperm may be further optimized through oral antioxidants [33].

In regards to Case 3, little is known about the pathophysiology of poor IVF-ICSI outcomes in Kartagener’s Syndrome. Given the rarity of the disease, most of the primary literature is based upon case reports [35]. It is assumed that Kartagener’s Syndrome results in longer transit times through the epididymis, which may result in greater oxidative damage, cell senescence, and loss of DNA integrity [36, 37]. An analysis by Cayan et al. demonstrated substantial improvement of sperm viability with testicular sperm in one patient, presumably bypassing the damage that may develop within the epididymis [38]. Additionally, Westlander and colleagues concluded that more reliable fertilization rates can be achieved with testicular sperm [37]. Future studies are needed to define a link between this rare syndrome and elevated oxidative stress and/or increased DNA fragmentation.

The optimal source of sperm for IVF-ICSI remains controversial [11, 39]. Two recent series have compared the results of IVF-ICSI between ejaculated and testicular sperm. Lu et al. found a slight advantage of ejaculated sperm in terms of fertilization rates and embryo quality within one center’s large experience [4]. Similarly, Gnoth and colleagues analyzed 337 IVF-ICSI cycles and found no difference in clinical outcomes between ejaculated and testicular sperm for men with cryptozoospermia [6]. Both studies were limited by a retrospective design with significant potential for selection bias. In each case a testicular sperm extraction was used only when sperm could not be reliably found within the ejaculate. Neither study compared IVF-ICSI outcomes between ejaculated and matched testicular derived sperm from the same individual. Additionally, these studies did not stratify for the cohort of men who have had repeated IVF-ICSI failure, a group that may benefit from testis-derived sperm [10, 11, 40, 41]. Our small case series documents a possible benefit for this population. It should be noted, however, that our study is also limited by a retrospective design. Additionally, the criteria used to select for PercBx was not standardized at our institution, and so one must consider a component of selection bias. Despite these limitations in study design, our data do suggest an indication for retrieving testicular sperm despite sperm availability within the ejaculate. Further studies are needed to confirm our observations.

## Conclusions

It remains to be elucidated if patients with recurrent IVF-ICSI failure have progressive deterioration of sperm quality, and therefore worse pregnancy outcomes, due to sperm passage through the male reproductive tract [26]. Larger studies are ultimately needed to substantiate these pilot investigations and preliminary observations. Until these data are obtained, for couples who undergo repeated unsuccessful cycles of IVF-ICSI with ejaculated sperm, a trial of freshly procured testicular sperm may offer an improved chance to achieve parenthood.

### Ethics approval

This study was approved by the Massachusetts General Hospital Institutional Review Board.

### Consent for publication

Not applicable

### Availability of data and material

All data leading to the conclusions of this paper are available in the results section of this manuscript.

## Abbreviations

DFI: DNA fragmentation index
FSH: follicle stimulating hormone
IVF: in vitro fertilization with conventional insemination
IVF-ICSI: in vitro fertilization and intracytoplasmic sperm injection
OAT: oligoasthenoteratospermia
PercBx: percutaneous testis biopsy
ROS: reactive oxygen species
SCSA: sperm chromatin structure assay
